# Profiling Humoral Immune Response Against Pre-Erythrocytic and Erythrocytic Antigens of Malaria Parasites Among Neotropical Primates in the Brazilian Atlantic Forest

**DOI:** 10.3389/fcimb.2021.678996

**Published:** 2021-05-13

**Authors:** Gabriela Maíra Pereira de Assis, Denise Anete Madureira de Alvarenga, Matheus de Oliveira Costa Pereira, Juan Camilo Sánchez-Arcila, Anielle de Pina Costa, Júlio César de Souza Junior, Ana Julia Dutra Nunes, Alcides Pissinatti, Silvia Bahadian Moreira, Leticia de Menezes Torres, Helena Lott Costa, Herlandes da Penha Tinoco, Valéria do Socorro Pereira, Irene da Silva Soares, Taís Nóbrega de Sousa, Francis Babila Ntumngia, John H. Adams, Flora Satiko Kano, Zelinda Maria Braga Hirano, Cláudio Tadeu Daniel-Ribeiro, Joseli Oliveira Ferreira, Luzia Helena Carvalho, Cristiana Ferreira Alves de Brito

**Affiliations:** ^1^ Grupo de Pesquisa em Biologia Molecular e Imunologia da malária, Instituto René Rachou/Fiocruz Minas, Belo Horizonte, Brazil; ^2^ Laboratório de Imunoparasitologia, Instituto Oswaldo Cruz (IOC), Fiocruz, Rio de Janeiro, Brazil; ^3^ Centro de Pesquisa, Diagnóstico e Treinamento em Malária (CPD-Mal), Fiocruz, Rio de Janeiro, Brazil; ^4^ Ambulatório de Doenças febris, Instituto Nacional de Infectologia (INI), Ambulatório de Doenças Febris Agudas Fiocruz, Rio de Janeiro, Brazil; ^5^ Centro Universitário Serra dos Órgãos (UNIFESO), Teresópolis, Brazil; ^6^ Centro de Pesquisas Biológicas de Indaial, Indaial, Brazil; ^7^ Fundação Universidade Regional de Blumenau (FURB), Blumenau, Brazil; ^8^ Programa de conservação do Bugio Ruivo, Joinville, Brazil; ^9^ Centro de Primatologia do Rio de Janeiro (CPRJ), Instituto Estadual do Ambiente (INEA), Guapimirim, Brazil; ^10^ Fundação de Parques Municipais e Zoobotânica (FPMZB), Belo Horizonte, Brazil; ^11^ Departamento de Análises Clínicas e Toxicológicas, Faculdade de Ciências Farmacêuticas, Universidade de São Paulo, São Paulo, Brazil; ^12^ Center for Global Health and Infectious Diseases Research, College of Public Health, University of South Florida, Tampa, FL, United States; ^13^ Laboratório de Pesquisa em Malária, IOC/Fiocruz, Rio de Janeiro, Brazil

**Keywords:** malaria, humoral response, neotropical primates, Atlantic forest, pre-erythrocytic antigen, erythrocytic antigens

## Abstract

Human malaria due to zoonotic transmission has been recorded in the Atlantic Forest, an extra-Amazonian area in Brazil, which are a challenge for malaria control. Naturally acquired humoral immune response against pre-erythrocytic and erythrocytic antigens of Neotropical primates (NP) was evaluated here to improve the knowledge about the exposure of those animals to the malaria transmission and support the identification of the potential reservoirs of the disease in the Atlantic Forest. Blood samples of 154 monkeys from three areas of the Atlantic Forest were used to identify IgG antibodies against peptides of the repeat region of the major pre-erythrocytic antigen, the circumsporozoite protein (CSP), of *Plasmodium vivax* (PvCSP), *Plasmodium brasilianum/Plasmodium malariae* (Pb/PmCSP), and *Plasmodium falciparum* (PfCSP) by ELISA. Antibodies against erythrocytic recombinant antigens of *P. vivax*, Apical membrane antigen 1 (PvAMA-1), Erythrocyte binding protein 2 (PvEBP-2) and domain II of Duffy binding protein (PvDBPII) were also evaluated. Parameters, such as age, sex, PCR positivity, and captivity, potentially associated with humoral immune response were analyzed. Eighty-five percent of NP had antibodies against at least one CSP peptide, and 76% against at least one *P. vivax* erythrocytic antigen. A high percentage of adults compared to non-adults were seropositive and showed increased antibody levels. Neotropical primates with PCR positive for *P. simium* had a significantly higher frequency of positivity rate for immune response against PvEBP-2, PvDBPII and also higher antibody levels against PvDBPII, compared to PCR negative NPs for this species. Monkeys with PCR positive for *P. brasilianum/P. malariae* showed higher frequency of seropositivity and antibody levels against Pb/PmCSP. Levels of antibodies against Pb/PmCSP, PvEBP-2 and PvDBPII were higher in free-living than in captive monkeys from the same area. All Platyrrhine families showed antibodies against CSP peptides, however not all showed IgG against erythrocytic antigens. These findings showed a high prevalence of naturally acquired antibodies against CSP repeats in all studied areas, suggesting an intense exposure to infected-mosquitoes bites of NP from all families. However, mainly monkeys of Atelidae family showed antibodies against *P. vivax* erythrocytic antigens, suggesting blood infection, which might serve as potential reservoirs of malaria in the Atlantic Forest.

## Introduction

Malaria remains an important public health problem despite of many efforts to control the disease around the World. According to the latest estimates of the World Health Organization, there were about 229 million human malaria cases and 409 000 deaths in 2019 (World Health Organization, 2020). Five *Plasmodium* species are more frequently associated with human infection, *Plasmodium falciparum*, *P. vivax*, *P. malariae*, *P. ovale* and *P. knowlesi*. The last species was recently described in a generalized zoonotic transmission in Southeast Asia (Cox-Singh, 2012). In Brazil, more than 150 000 cases of malaria were registered in 2019 (World Health Organization, 2020). Malaria is endemic almost exclusively within the Brazilian Amazon region, caused mainly by *P. vivax* (89%), followed by *P. falciparum* (11%) and with few notified cases of *P. malariae* (< 1%) (Ministerio da Saude, 2020). Nevertheless, 732 malaria cases were registered in 2018 in the extra-Amazon Region, including the Atlantic Forest region. Most of the cases in this region were diagnosed as *P. vivax* (de Pina-Costa et al., 2014). Recently, Brasil et al. reported that cases from an outbreak in the Atlantic Forest of Rio de Janeiro state, initially diagnosed as *P. vivax* infection, were in fact caused by *Plasmodium simium*, a neotropical primate (NP) parasite (Brasil et al., 2017).

In the Brazilian forests, there are two malaria parasites primarily infecting primates: *Plasmodium brasilianum* and *Plasmodium simium*. *P. simium* infects primates of the Atlantic Forest from South and Southeastern Brazil of Atelidae, Cebidae and Pitheciidae families (da Fonseca, 1951; Deane, 1992; de Alvarenga et al., 2015). *Alouatta guariba clamitans* (southern brown howler monkeys) is the species most frequently infected by *P. simium* (Deane, 1992; de Alvarenga et al., 2015; Abreu et al., 2019). *P. brasilianum* infects primates of all neotropical primate families distributed from Central America to the South of Brazil (Seidelin, 1912; Deane, 1992; Lourenço-de-Oliveira and Deane, 1995; Lalremruata et al., 2015; Alvarenga et al., 2017). Of importance, *P. simium* and *P. brasilianum* are morphological, genetic, and immunologically similar to human *Plasmodium* species, *P. vivax* and *P. malariae*, respectively (Coatney, 1971; Seed, 1976; Cochrane et al., 1985; Barnwell, 1986; de Arruda et al., 1989; Deane, 1992; Goldman et al., 1993; Escalante et al., 1995; Escalante et al., 1998; Fandeur et al., 2000; Volney et al., 2002; Tazi and Ayala, 2011; de Alvarenga et al., 2015; Lalremruata et al., 2015; Brasil et al., 2017). Brasil et al. and Lalremruata et al. molecularly characterized *P. simium* and *P. brasilianum* infections in humans and NPs, indicating their zoonotic transmission and reinforcing that non-human primates may act as malaria reservoirs (Lalremruata et al., 2015; Brasil et al., 2017). Mourier et al. showed the close similarity of whole genomes of *P. simium* from human and monkeys, and comparing the genes involved in the erythrocyte invasion identified a possible adaptation of the parasite for human and monkey infections in the Brazilian Atlantic Forest and reinforce the hypothesis of zoonotic transmission of *P. simium/P. vivax* (Mourier et al., 2019). In this context, zoonotic malaria constitutes a major challenge for malaria elimination.

Considering the genetic proximity between *P. simium* and *P. vivax*, and between *P. brasilianum* and *P. malariae*, the study of these species contributes to understanding their evolution in the America region. However, there are only a few studies of monkeys naturally infected by *P. simium* and *P. brasilianum* and even a smaller number of studies of the immune response in these animals (Duarte et al., 2006; Yamasaki et al., 2011; Costa et al., 2014; Monteiro et al., 2020). Thus, the identification of species from different families of NPs will enable a better understanding of these species as potential malaria reservoirs and whether these *Plasmodium* species in their natural hosts showed a similar immune response compared to human malaria.

The present study comprises an unprecedented study of malaria antibodies, using both *Plasmodium* pre-erythrocytic and different erythrocytic stage antigens in NP from distinct areas of the Brazilian Atlantic Forest.

## Materials and Methods

### Characteristics of Studied Areas and Non-Human Primate Blood Samples

Samples were obtained from 154 NPs belonging to all families from three areas (Indaial/SC, Joinville/SC, and Guapimirim/RJ) of the Atlantic forest from South and Southeastern Brazil (**Supplementary Table 1**). Our group studied these areas and showed a distinct epidemiologic profile for malaria in NPs (Costa et al., 2014; de Alvarenga et al., 2018; Nunes et al., 2020). Blood was drawn from the femoral or brachial vein using tubes with anticoagulant (5% EDTA). After the collection, blood samples were centrifuged at 1500 *x g* for 10 min at room temperature and plasma stored at -20°C. Information about sex and age group from each animal were collected (**Table 1**). The age, estimated as proposed by Carpenter, was used to categorize the animals as non-adults (juveniles) and adults (including also subadults) (Carpenter, 1965).

**Table 1:** Characteristics of monkeys from each studied area.

Characteristic	Studied area (Municipality/State)			Total
	Indaial/SC	Joinville/SC	Guapimirim/RJ	
	N = 75	N = 39	N = 40	N = 154
**Sex ratio male:female**	1.27:1	0.95:1	1:1.22	1.05:1
**Ratio Adult: Non-adult** *^a^*	4.35:1	5.5:1	40:0	6.7:1
**Ratio Captive: Free-living**	5.25:1	0:39	40:0	2.01:1
**Families**				
Atelidae	75 (100%)*^b^*	39 (100%)*^b^*	8 (20%)*^c^*	122 (79.2%)
Aotidae	0	0	1 (2.5%)*^d^*	1 (0.6%)
Callitrichidae	0	0	4 (10%)*^e^*	4 (2.6%)
Cebidae	0	0	24 (60%)*^f^*	24 (15.6%)
Pitheciidae	0	0	3 (7.5%)*^g^*	3 (1.9%)
**Positivity by PCR*^h^***	10 (13%)	26 (67%)	13 (32%)	59 (32%)

Results are expressed in absolute numbers and percentages in parentheses. ^a^Age was estimated according to Carpenter, 1965. Studied species: ^b^Alouatta g. clamitans, ^c^Alouatta g.clamitans, Alouatta caraya, Ateles paniscus, Brachyteles arachnoides; ^d^Aotus nigriceps; ^e^Mico humeralifer, Leontopithecus chrysomelas, Leontopithecus rosalia, Saguinus midas; ^f^Cebus sp., Sapajus robustus, Sapajus xanthosternus; ^g^Cacajao melanocephalus, Callicebus personatus. ^h^Molecular diagnosis of Plasmodium sp. infection was previously performed by our group (Costa et al., 2014; de Alvarenga et al., 2015; Alvarenga et al., 2017; de Alvarenga et al., 2018; Nunes et al., 2020) according to Snounou et al. (1993).

**Figure 1:**
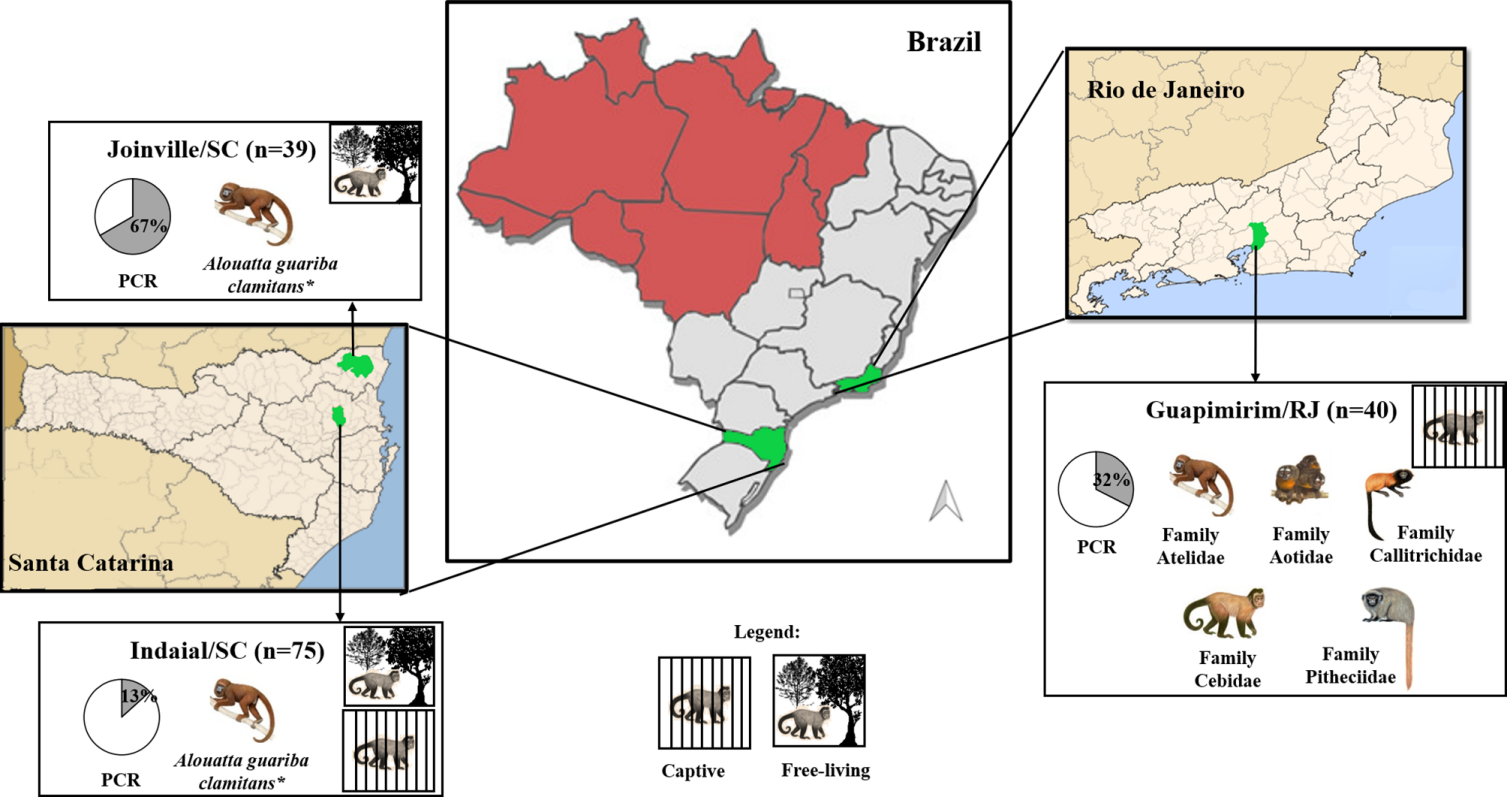
Geographical distribution of the three studied areas and characteristics of studied neotropical primates. Captive or free-living monkey pictures represent species/families of NP from each studied area. The pie graph represents the proportion (%) of positivity (gray) for *Plasmodium* by PCR (Snounou et al., 1993; de Alvarenga et al., 2018). Malaria endemic areas (red) and study areas (green). *Alouatta guariba clamitans is the only species present.

The first study area was in the municipality of Indaial (26°53’52” S/49°13’54” W) located in Santa Catarina state, South Brazil. In this municipality is located the Centre for Biological Research of Indaial (CEPESBI) (IBAMA register number 1/42/98/000708-90), a conservation unit for southern brown howler monkey located in the Valley of Itajaí in the Atlantic Forest (**Figure 1**). Samples from 63 captive *Alouatta guariba clamitans* were collected between 2008 and 2019. Samples from 12 free-living NP captured in the Geisler Mountain in Indaial or attended in the veterinary hospital of University Regional of Blumenau (FURB), from different areas of Santa Catarina state were also collected (**Supplementary Table 1**). Eighty-one percent of animals were adults (**Table 1**). *Plasmodium* infection was previously identified by our group in 13% of animals by polymerase chain reaction (PCR) (Snounou et al., 1993; Costa et al., 2014; de Alvarenga et al., 2018).

The second area was in the city of Joinville, located also in Santa Catarina state, South Brazil (26°14’41.78” S/48°53’02.78” W), in an Atlantic forest conservation area located on a private property (**Figure 1**). Samples of 39 free-living howler monkeys were collected from July to December in 2015 and 2017 (for more details about capture see Ref. Nunes et al., 2020). The capture has prioritized adults, resulting in 85% of the monkeys (**Table 1**). *Plasmodium* infection was previously identified in 67% of studied animals that were infected with *P. simium* and/or *P. brasilianum* by PCR (Nunes et al., 2020).

The third area was in the municipality of Guapimirim, located in Rio de Janeiro state, Southeast Brazil (22°29’18.28” S/42°54’48.43” W) (**Figure 1**). This municipality is home to the Primate Centre of Rio de Janeiro (CPRJ), a conservation unit for wild monkeys on the Serra dos Órgãos, in the Atlantic Forest, about 100 km from the city of Rio de Janeiro. Samples from 40 captive adult monkeys belonging to all Platyrrhini families were collected from January 2011 to October 2019 (**Table 1**). *Plasmodium* infection was previously identified in 32% of animals by PCR (de Alvarenga et al., 2015; Alvarenga et al., 2017).

### Ethics Statement

Capture and biological samples collection were performed according to the Brazilian guidelines and regulations and were approved by the Fiocruz Research Ethical Committee (CEUA license L037/2016) and by the Brazilian Ministry of Environment (SISBIO numbers 43375-4, 43375-6, 54707-137362-2, 52472-1, 28953-1).

### Enzyme-Linked Immunosorbent Assay

#### Pre-Erythrocyte Antigen

Synthetic peptides representing the repeated immunodominant epitope of the major pre-erythrocytic antigen, the circumsporozoite protein (CSP), were used for the detection of immunoglobulin G (IgG) antibodies. All three variant sequences of *P. vivax* (PvCSP) were used: *P. vivax* “classic” VK210 (Pvc) DGQPAGDRAAGQPAG-(DRADGQPAG)_2_; *P. vivax* VK247 (Pvk) (ANGAGNQPG)_3_-ANGAGN; and *P. vivax*-like (Pvl) (APGANQEGGAA)_3_. CSP peptides from *P. falciparum* (NANP)_8_ and *P. malariae/P. brasilianum* (GNAA)_2_-GNDA(GNAA)_4_ were also used. *P. brasilianum* and *P. malariae* share identical CSP repeat sequences, it was named here Pb/PmCSP (Guimarães et al., 2012; Pereira et al., 2018).

The enzyme-linked immunosorbent assay (ELISA) for the detection of antibodies to the CSP was performed, as previously described (Duarte et al., 2006; Pereira et al., 2018), with modifications. Briefly, MaxiSorp™ plates (Nunc, Carlsbad, CA, USA) were coated with 10 μg/mL of the CSP synthetic peptides diluted in PBS (Phosphate Buffer Solution), and incubated for 12-16 hours at 4°C. Individual plasma samples were incubated at 1:50 dilution in PBST (PBS + 0.05% Tween 20) plus 2.5% Bovine Serum Albumin (BSA, Sigma-Aldrich, St Louis, MO, USA), for one hour at 37°C. Antibodies were detected by anti-*Macaca mulatta* IgG-peroxidase conjugated (Sigma-Aldrich) at 1:3000 dilution in PBST plus 2.5% BSA, and incubated one hour at 37°C. In each well was added 50 µL of the solution with 20 μg O-phenylenediamine dihydrochloride (Sigma-Aldrich, USA) diluted in 50 mL of sodium citrate buffer (0.1 M pH 5.0) in the presence of 40 µL hydrogen peroxide and left in dark for 20 min at 37°C. Reaction development was interrupted by adding 25 µL of 2 M H_2_SO_4_ in each well. The optical densities (ODs) were evaluated on an ELISA reader (Spectra Max 340PC 384, Molecular Devices) at 490 nm. Results were expressed as reactivity index (RI), which was calculated by dividing the mean of OD values of tested samples by the mean plus three standard deviations (SDs) of negative control samples (sera of three *Alouatta g. clamitans* from Fundação de Parques Municipais e Zoobotânica at Belo Horizonte in Minas Gerais state, Brazil, a non-transmission malaria area). This species was chosen because it represents 77% of the studied animals herein. Samples with a RI greater than 1 were considered positive. For comparisons with immune response against erythrocytic antigens, samples were considered positive for PvCSP when they showed RI >1 for any of three CSP repeats (VK210, VK247 or *P. vivax*-like).

#### *P. vivax* Erythrocytic Antigens

Conventional enzyme-linked immunoassays (ELISA) for total IgG antibodies against *P. vivax* recombinant proteins, domain II of Duffy binding protein (PvDBPII) from Sal-1 strain, Apical membrane antigen 1 (PvAMA-1) and Erythrocyte binding protein 2 (PvEBP-2) were carried out as previously described (Cerávolo et al., 2005; Kano et al., 2012; Pires et al., 2018) with modifications. Our research group has been working for years on the production of recombinant proteins for the study of the human immune response against *P. vivax* antigens in endemic areas for malaria. Therefore, we chose to use only *P. vivax* erythrocytic antigens due to their availability in our laboratory, considering the homology between *P. vivax* and *P. simium* and the high prevalence of *P. simium* in the Atlantic Forest. Recombinant proteins were used at a final concentration of either 1.5 µg/mL (PvEBP-2 and PvAMA-1) or 3 µg/mL (PvDBPII). Serum samples were used at 1:100 dilution, and peroxidase-conjugated anti-IgG of *Macaca mulatta* was used as secondary antibody at 1:80 000 dilution (Sigma-Aldrich) in PBST plus 3% skimmed milk powder, and incubated for one hour at 37°C. For each protein, the results were expressed as ELISA reactivity index (RI), values of RI > 1.0 were considered positive.

### Statistical Analysis

Analyzes were done using GraphPad Prism version 7.0 (GraphPad Software, Inc., San Diego, CA, USA) and R version 4.0.2 (Vienna, Austria) (R Core Team, 2020). The distribution of parameters was determined by the Kolmogorov Smirnov and Shapiro-Wilk tests. The differences between medians or means of two groups were verified using the T-test or the Mann-Whitney test, according to the data distribution. The comparisons between more than two groups were performed using the Analysis of Variance (ANOVA) or Kruskal-Wallis test, followed by Tukey or Dunn’s *post hoc* test, according to the data distribution. The association between categorical variables was assessed using the Chi-square test (X^2^) or Fisher test. Heatmaps for antibody levels against *Plasmodium* antigens were created with ComplexHeatmap (Gu et al., 2016). The intersections of responders to PvCSP and *Plasmodium vivax* erythrocytic antigens were plotted using the “UpSetR” R package (v1.4.0) (Conway et al., 2017). Analyzes of correlation between antibody levels of responders were assessed with Sperman’s rank correlation. In all analyzes, a significance level of 5% was considered, i.e., values of *P* < 0.05.

## Results

### Naturally Acquired Antibody Responses Against CSP Peptides

**Figure 2:**
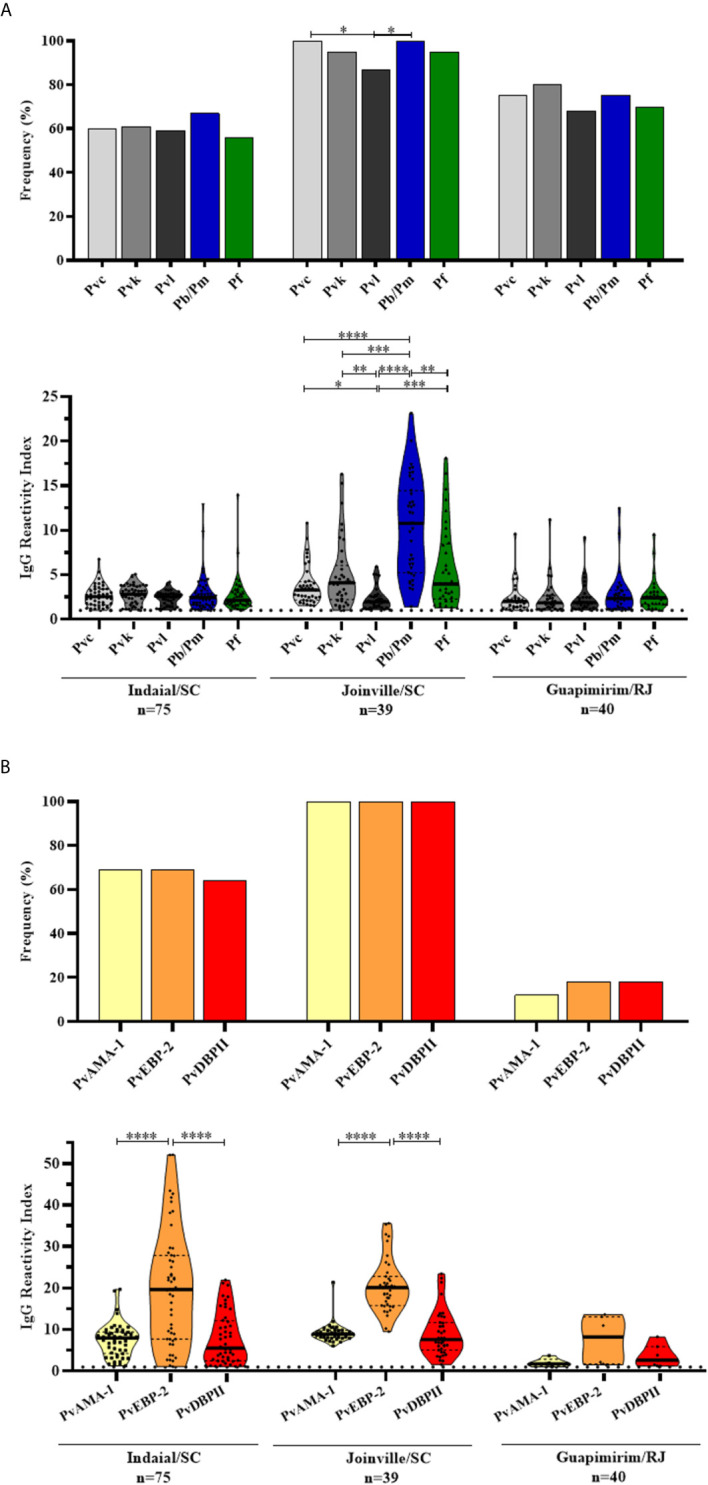
Prevalence and levels of antibodies against *Plasmodium* antigens in neotropical primates. Frequency and reactivity index (RI) of IgG antibodies against **(A)** CSP peptides and **(B)** *Plasmodium vivax* erythrocytic antigens among NP from Indaial/SC, Joinville/SC, and Guapimirim/RJ. RI > 1 was considered positive (dotted line). The filled bars indicate the percentage of NP with RI > 1 (**A**, **B** top). Data expressed as individual RI values (dots) and median with the interquartile range (boxes) (**A**, **B** botton). Pvc: *P. vivax* CSP VK210 (light gray); Pvk: *P. vivax* CSP VK247 (gray); Pvl: CSP *P. vivax*-like (dark gray); Pb/Pm: *P. brasilianum/P. malariae* (blue); Pf: *P. falciparum* (green); PvAMA-1: *P. vivax* Apical membrane antigen *–* 1 (yellow); PvEBP-2: *P. vivax* Erythrocyte Binding Protein 2 (orange); PvDBPII: *P. vivax* Duffy Binding Protein region II (red). In the bottom of each graph is shown the numbers (n) of NP included in each area. Differences statistically significant were indicated by asterisk (*P < 0.05, **P < 0.01, ***P < 0.001, ****P < 0.0001).

Overall, 85% (131/154) of NP showed IgG response against at least one of the five CSP peptides (Pvc: *P. vivax* VK210; Pvk: *P. vivax* VK247; Pvl: *P. vivax*-like, *P. brasilianum/P. malariae* and *P. falciparum*). The highest frequency of positive animals for any CSP peptide was identified in Joinville/SC (100%), followed by Guapimirim/RJ (90%) and Indaial/SC (75%) (**Figure 2A** top). The frequency of positive NP was significantly higher against Pb/PmCSP and PfCSP only in Joinville/SC (*P* < 0.05, Chi-square test test).

The levels of antibodies were low and similar among all peptides in Indaial/SC and Guapimirim/RJ. In Joinville/SC, antibody levels were significantly highest against Pb/PmCSP (RI median = 10.78) than against other CSP peptides (*P* < 0.01, Kruskal-Wallis test) (**Figure 2A** bottom).

### Naturally Acquired Antibody Responses Against *Plasmodium vivax* Erythrocytic Antigens

Significant levels of IgG against at least one of the three erythrocytic antigens were identified in 76% (119/154) of studied NP. The seroprevalence for any erythrocytic antigen was highest for Joinville/SC animals (100%), followed by Indaial/SC (90%) and Guapimirim (32%), and similar among antigens in each studied area (**Figure 2B** top).

Antibody titers against PvEBP-2 were higher than PvAMA-1 and PvDBPII in Indaial/SC (RI median: PvEBP-2 = 19.65, PvAMA-1 = 7.98, and PvDBPII = 5.58, *P* < 0.0001, Kruskal-Wallis test) and in Joinville/SC (RI median: PvEBP-2 = 20.08 PvAMA-1 = 8.91, and PvDBPII = 7.59, *P* < 0.0001, Kruskal-Wallis test) (**Figure 2B** bottom).

### Factors Potentially Associated With Seropositivity and Antibody Levels Against CSP and *P. vivax* Erythrocytic Antigens

**Figure 3:**
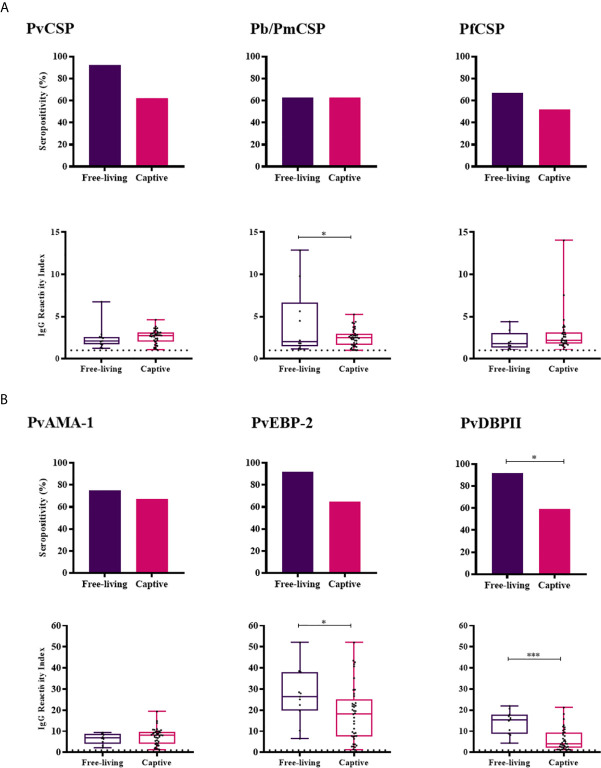
Prevalence and levels of antibodies against *Plasmodium* antigens in Free-living and Captive animals from Indaial/SC. Seropositivity and reactivity index (RI) of IgG antibodies against **(A)** CSP peptides and **(B)** *Plasmodium vivax* erythrocytic antigens. RI > 1 was considered positive (dotted line). The filled bars indicate the percentage of NP with RI > 1 (**A**, **B** top). Data expressed as individual RI values (dots) and median with the interquartile range (boxes) (**A**, **B** botton). PvCSP: CSP repeats representing *P. vivax* CSP variants (VK210, VK247, and *P. vivax-like*); Pb/PmCSP: CSP repeat of *P. brasilianum/P. malariae*; PfCSP: CSP repeat of *P. falciparum*; PvAMA-1: *P. vivax* Apical membrane antigen– 1; PvEBP-2: *P. vivax* Erythrocyte Binding Protein 2; PvDBPII: *P. vivax* Duffy Binding Protein region II. Free-living: purple; Captive: pink. Differences statistically significant were indicated by asterisk (*P < 0.05; ***P < 0.001).

Free-living monkeys showed a higher IgG response against Pb/PmCSP, PvEBP-2 and PvDBPII when compared to the captive monkeys (*P* < 0.05, Mann-Whitney test) (**Figure 3**). The frequency of positivity in free-living monkeys was also higher for PvDBPII. The comparison was possible only in Indaial/SC because only in this area captive and free-living monkeys were under similar transmission pattern.

**Figure 4:**
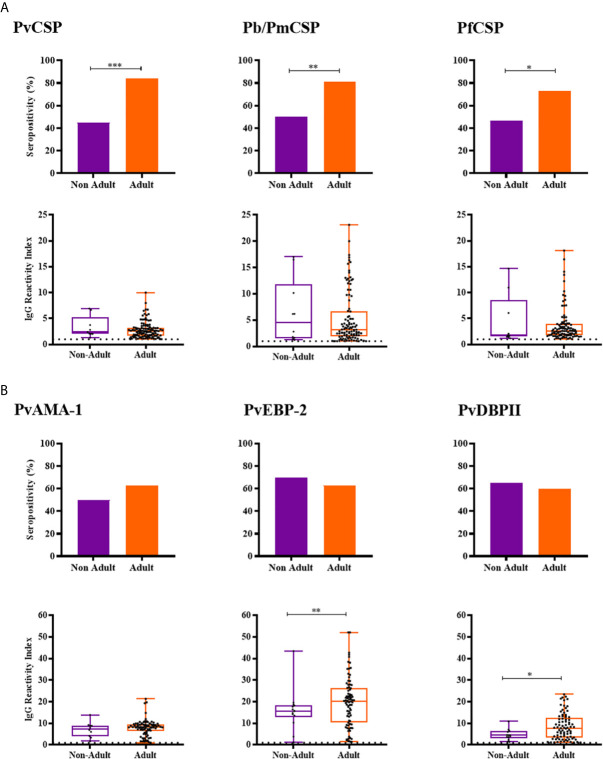
Prevalence and levels of antibodies against *Plasmodium* antigens according to age of neotropical primates. **(A)** Seropositivity and reactivity index (RI) of IgG antibodies against CSP peptides. **(B)** Seropositivity and RI of IgG antibodies against *Plasmodium vivax* erythrocytic antigens. RI > 1 was considered positive (dotted line). The filled bars indicate the percentage of NP with RI > 1 (**A**, **B** top). Data expressed as individual RI values (dots) and median with the interquartile range (boxes) (**A**, **B** botton). Age estimated according to Carpenter, 1965, and categorized in non-Adult (light purple) and Adult (orange). PvCSP: CSP repeats representing *P. vivax* CSP variants (VK210, VK247, and *P. vivax-like*); Pb/PmCSP: CSP repeat of *P. brasilianum/P. malariae*; PfCSP: CSP repeat of *P. falciparum*; PvAMA-1: *P. vivax* Apical membrane antigen– 1; PvEBP-2: *P. vivax* Erythrocyte Binding Protein 2; PvDBPII: *P. vivax* Duffy Binding Protein region II. Differences statistically significant were indicated by asterisk (*P < 0.05; **P < 0.01; ***P < 0.001).

Adult NPs had a higher IgG positivity rate than non-adults against CSP peptides from the three *Plasmodium* species (Pv, Pb/Pm and Pf) and higher levels of antibodies against PvEBP-2 (RI median = 20.24 and 15.69, respectively; *P* = 0.0087, Mann-Whitney test) and PvDBPII (RI median = 7.727 and 4.454, respectively; *P* = 0.0253, Mann-Whitney test) (**Figure 4**). Seroprevalences and antibody titers were similar between sexes for CSP peptides and erythrocytic antigens (*P* > 0.05, Fisher test and Mann-Whitney test) (**Supplementary Figure 1**).

**Figure 5:**
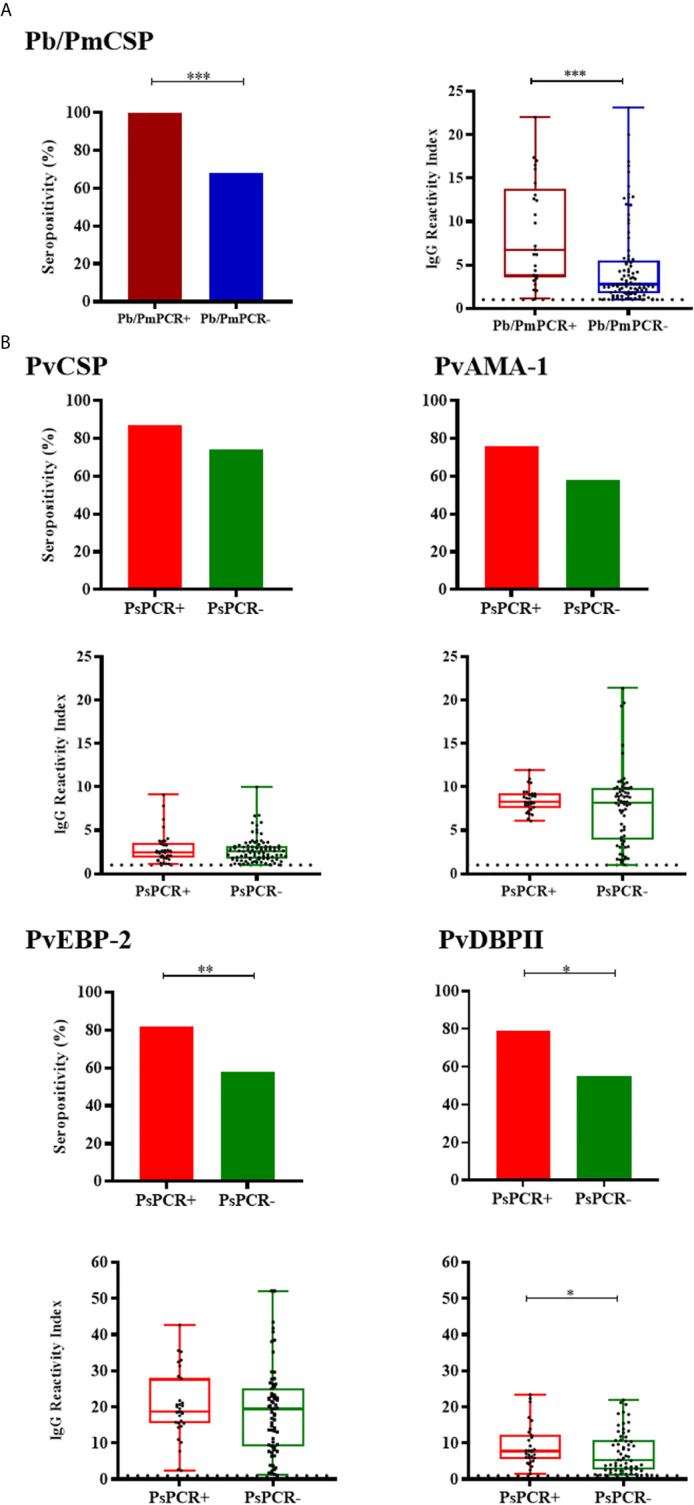
Prevalence and levels of antibodies against *Plasmodium* antigens according to PCR positivity for *Plasmodium* species. **(A)** Seropositivity and reactivity index (RI) of IgG antibodies against *P. brasilianum/P. malariae* CSP peptide in monkeys with PCR positive for *P. brasilianum/P. malariae* (Pb/PmPCR+, dark red) or negative (Pb/PmPCR-, blue). **(B)** Seropositivity and RI of IgG antibodies against *P. vivax* CSP and erythrocytic antigens in monkeys with PCR positive for *P. simium* (PsPCR+, red) or negative (PsPCR-, green). RI > 1 was considered positive (dotted line). The filled bars indicate the percentage of NP with RI > 1 (**A**, **B** top). Data expressed as individual RI values (dots) and median with the interquartile range (boxes) (**A**, **B** botton). Pb/PmCSP: CSP repeat of *P. brasilianum/P. malariae*; PvCSP: CSP repeats representing *P. vivax* CSP repeats (VK210, VK247, and *P. vivax*-like); PvAMA-1: *P. vivax* Apical membrane antigen – 1; PvEBP-2: *P. vivax* Erythrocyte Binding Protein 2; PvDBPII: *P. vivax* Duffy Binding Protein region II. Differences statistically significant were indicated by asterisk (*P < 0.05; **P < 0.01; ***P < 0.001).

Among NPs with PCR positive for *P. brasilianum/P.malariae* (Pb/PmPCR+), frequency of responders and levels of antibodies against Pb/PmCSP were higher than in negative monkeys for this *Plasmodium* species (Pb/PmPCR-) (seroprevalences: 100% and 68%, respectively, *P* = 0.0003, Fisher test; antibody levels: RI median = 6.76 and 2.75, respectively, *P* = 0.0002, Mann-Whitney test) (**Figure 5A**). Frequencies of seropositivity against PvEBP-2 and PvDBPII were higher in NPs with PCR positive for *P. simium* (PsPCR+) as compared to NPs with PCR negative for *P. simium* (PsPCR-) (**Figure 5B**). PsPCR+ monkeys also showed higher levels of antibodies against PvDBPII than PsPCR- (RI median = 7.71 and 5.34, respectively; *P* = 0.0479, Mann-Whitney test). Comparing antibody response in PCR+ for any species of *Plasmodium* and PCR- monkey groups, the positivity rate to Pb/PmCSP and antibody levels against PvDBPII and PvEBP-2 were higher in PCR+ monkeys (**Supplementary Figure 2**).

### Naturally Acquired Antibody Responses Against *Plasmodium* spp. Antigens Among Neotropical Primate Families

**Figure 6:**
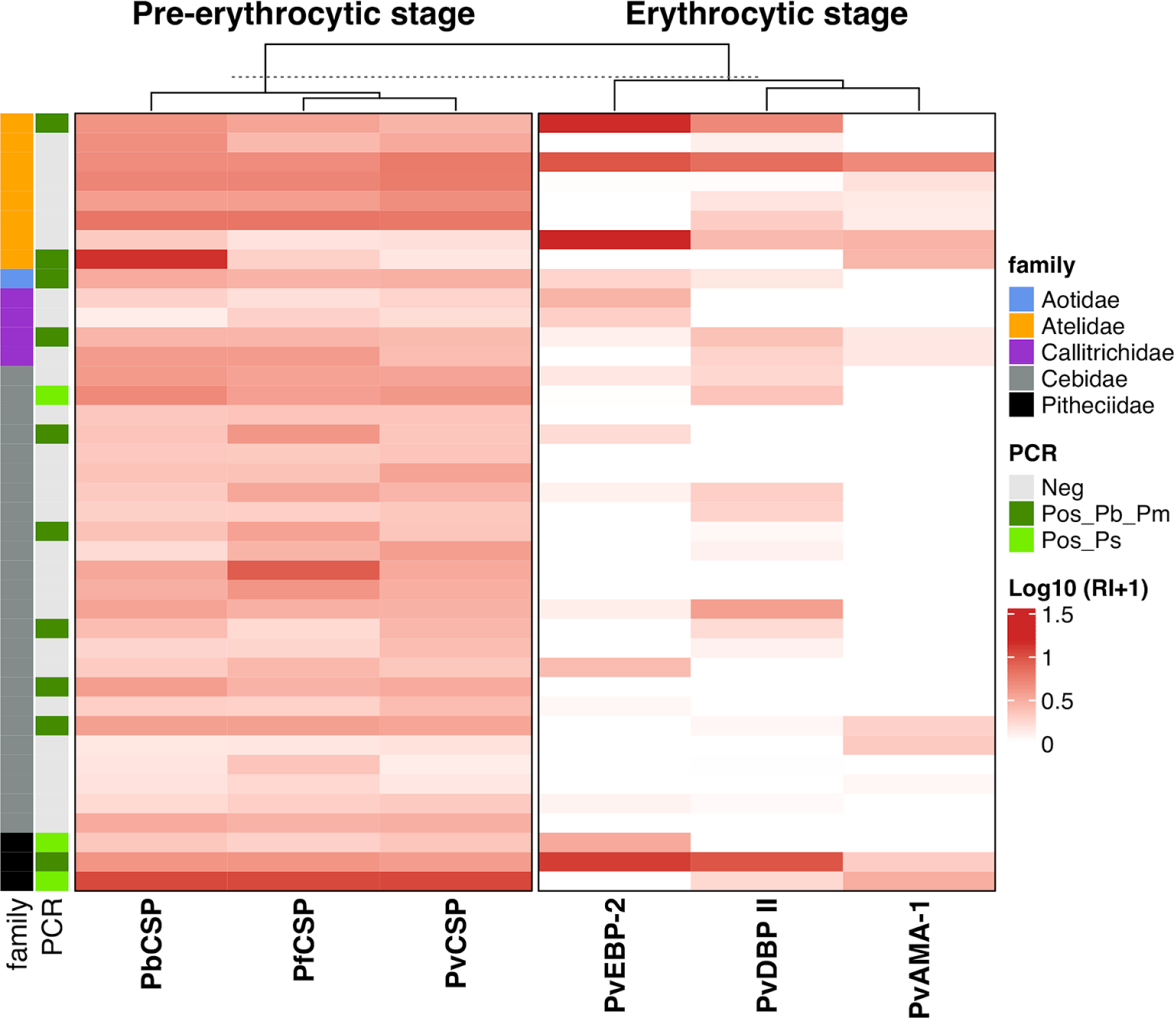
Heatmap of antibody levels against *Plasmodium* antigens among monkeys from Platyrrhni families. IgG antibody levels against CSP repeats from different *Plasmodium* species and *Plasmodium vivax* erythrocytic antigens among Platyrrhni families from Guapimirim/RJ. Antibody response IgG was expressed as Log10 Reactivity Index (RI+1). RI values increase with more intense red color. PvCSP: CSP repeats representing *P. vivax* CSP repeats (VK210, VK247, and *P. vivax*-like); Pb/PmCSP: CSP repeat of *P. brasilianum/P. malariae*; PfCSP: CSP repeat *of P. falciparum*; PvAMA-1: *P. vivax* Apical Membrane antigen – 1; PvEBP-2: *P. vivax* Erythrocyte Binding Protein 2; PvDBPII: *P. vivax* Duffy Binding Protein region II. Animals positive for *Plasmodium* by PCR (light green square – *P. simium*; dark green square - *P. brasilianum/P. malariae*) or negative – grey square.

All animals from Atelidae family showed antibodies against at least one of the three CSP peptides (Pv, Pb/Pm and Pf) and half of them also showed antibodies against erythrocytic antigens (**Figure 6**). Most animals in this family had medium to high levels of antibodies. A unique specimen of Aotidae family showed low/medium levels of IgG antibodies against the three CSP peptides but does not show antibodies against any erythrocytic antigens of *P. vivax*. In Callitrichidae family, half of the animals had low to high levels of antibodies against all three CSP peptides and two animals showed low levels of antibodies against only one erythrocytic antigen (**Figure 6**). Ninety-two percent of animals from Cebidae family showed antibodies against at least one CSP peptide. However, only 17% of NP from this family showed antibodies against any erythrocytic antigen, mostly in lower levels. All specimens of Pitheciidae family showed antibodies against, at least, one CSP peptide and one erythrocytic antigen, generally in high levels (**Figure 6**). All these analyzes were performed with animals from Guapimirim/RJ because the other areas had species only from Atelidae family (**Table 1**).

### Association Between the Immune Response Against PvCSP and Against Erythrocytic Antigens of *Plasmodium vivax*

**Figure 7:**
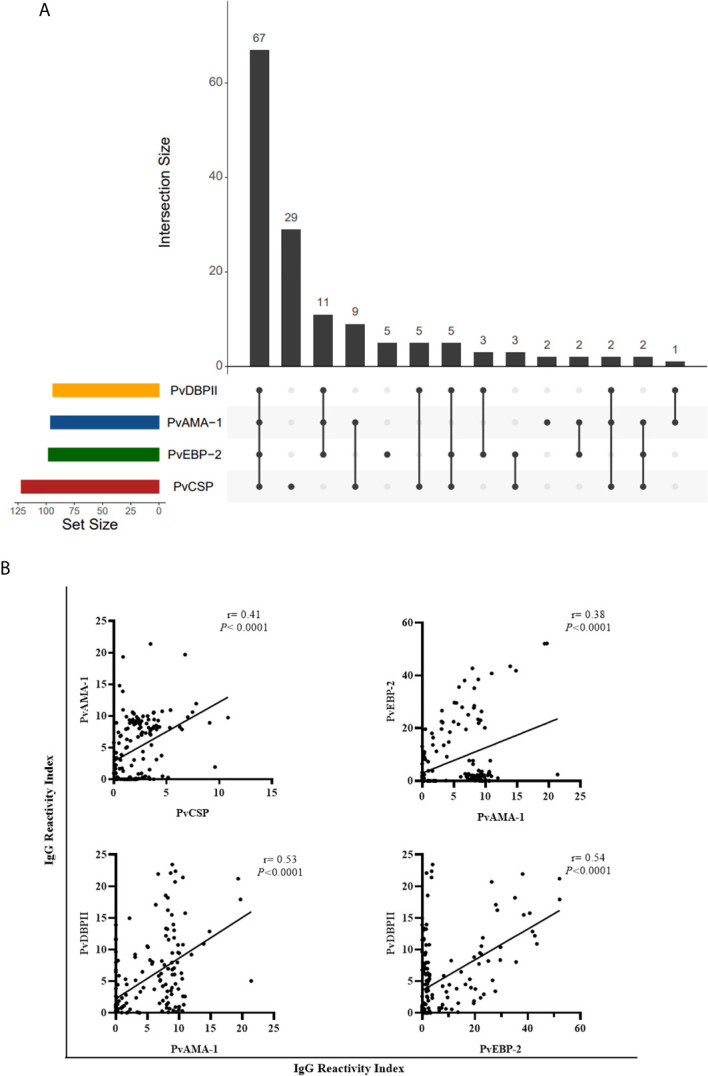
Association between responder monkeys against pre-erythrocytic and erythrocytic antigens of *Plasmodium vivax.* **(A)** UpSetR plot of responders to PvCSP and *P. vivax* blood stage antigens among all responder animals. **(B)** Significant correlation between reactivity index of responders to PvCSP and *P. vivax* blood stage antigens and among RI of blood stage antigens. Data expressed the value of r (Sperman’s rack correlation). PvCSP: CSP repeats representing *P. vivax* CSP repeats (VK210, VK247, and *P. vivax*-like); PvDBPII: *P. vivax* Duffy Binding Protein region II; PvAMA-1: *P. vivax* Apical Membrane antigen – 1; PvEBP-2: *P. vivax* Erythrocyte Binding Protein 2.

Ninety-three of responder NPs showed antibodies against PvCSP and any erythrocytic antigen and 67 of responder NPs showed antibodies against all studied antigens (pre-erythrocytic and erythrocytic) (**Figure 7A**). Significant positive correlations (*P* < 0.0001, Sperman’s test) were identified between antibody levels against PvCSP and PvAMA-1 (r = 0.41), PvAMA-1 and PvEBP-2 (r = 0.38), PvAMA-1 and PvDBPII (r = 0.53), and PvEBP-2 and PvDBPII (r = 0.54) (**Figure 7B**). This comparison was also performed grouping animals in PCR+ and PCR-, the only significant difference among PCR+ animals was the correlation between immune response against DBPII and EBP-2 (r= 0.44) (data not shown).

## Discussion

Neotropical primates infected with *P. simium* and *P. brasilianum* in the Brazilian Atlantic Forest have been largely reported (Deane, 1992; Duarte et al., 2006; Yamasaki et al., 2011; Costa et al., 2014; de Alvarenga et al., 2015; Alvarenga et al., 2017; Monteiro et al., 2020; Nunes et al., 2020). However, the humoral immune response of NPs naturally exposed to *Plasmodium* spp. transmission in this biome is still poorly understood. This is the first study that evaluates the humoral response of neotropical primates from different areas of the Atlantic forest against pre-erythrocytic and erythrocytic antigens of malaria parasites. The detection of antibodies against CSP peptides, the major pre-erythrocytic antigen covering the entire sporozoite surface, was evaluated to access the immune responses of NPs exposure to infected mosquito bites. Additionally, the presence of IgG against erythrocytic *P. vivax* antigens was evaluated to identify immune response in potentially well-established *Plasmodium* blood infection.

In the three studied areas, most neotropical primates had antibodies against *P. vivax* CSP variants, in slightly higher frequency against *P. vivax* VK210 and VK247. These CSP variants were the most frequent for *P. vivax* and the only ones described for *P. simium* (Goldman et al., 1993; Oliveira-Ferreira et al., 2004; Santos et al., 2019). Herein, NPs also showed antibodies against *P. brasilianum/P. malariae* CSP, as previously reported for NPs from Atlantic Forest in the São Paulo state (Duarte et al., 2006; Yamasaki et al., 2011). High seroprevalence against *P. falciparum* CSP was observed here, in concordance to previous reports of NP from other states (Duarte et al., 2006; Yamasaki et al., 2011). Although, all our NP samples were negative for *P. falciparum* infection, and only few descriptions of *P. falciparum* infected humans and mosquitoes have been described in the Atlantic forest area (Cerutti et al., 2007; Maselli et al., 2014; Laporta et al., 2015). The occurrence of a certain cross-reactivity degree among CSP repeats from different *Plasmodium* species, and also among *P. falciparum* CSP and asparagine-rich proteins from asexual parasite stage of other *Plasmodium* species in humans as well as non-human primates has been reported (Hope et al., 1984; Cochrane et al., 1990; Cochrane and Maracic, 1991; Volney et al., 2002; Hall et al., 2019). However, the association of seroprevalence against CSP and PCR positivity for specific *Plasmodium* species (*P.vivax/P.simium* and *P.brasilianum/P.malariae*) argued against the cross-reactivity. Therefore, further experiments are still needed to confirm the circulation of *P. falciparum* in the Atlantic Forest and immune response studies using erythrocytic antigens of *P. falciparum* and other *Plasmodium* species, such as *P. malariae/P.brasilianum*.

Based on the high similarity between *P. simium* and *P. vivax*, recombinant proteins of three leading erythrocytic stage surface proteins of *P. vivax*, PvAMA-1, PvDBPII, and PvEBP-2, were used here to evaluate the antibody response of NPs from Atlantic forest. In general, 76% of NP showed antibodies against *P. vivax* erythrocytic antigens. PvEBP-2 was the most frequently detected antigen and with the highest levels of antibodies. Recently, PvEBP-2 was suggested as a novel ligand for a potential alternative invasion pathway of Duffy positive reticulocytes (Hester et al., 2013; Ntumngia et al., 2016). Papua New Guinean children had IgG against PvEBP-2 correlated with protection against clinical malaria (França et al., 2017; He et al., 2019). The first evaluation of antibodies against *P. vivax* EBP-2 in non-human primates was undertaken here. High frequency of seroprevalence (63%) against MSP-1, another erythrocytic antigen, from different *Plasmodium* species was also demonstrated for animals from Atlantic forest in São Paulo and Santa Catarina states (Yamasaki et al., 2011; Monteiro et al., 2020). This seroprevalence was higher for free-living animals from the Atlantic forest than for animals from Amazon or Cerrado biome regions (Monteiro et al., 2020). These findings corroborate our suggestion of a high circulation of *Plasmodium* among NP in the Atlantic Forest area (Deane, 1992; de Pina-Costa et al., 2014; Brasil et al., 2017). Significant correlations of the humoral response against pre-erythrocytic and erythrocytic antigens found herein have been also described in humans (França et al., 2017; He et al., 2019).

Free-living animals showed significantly higher levels of antibodies against Pb/PmCSP, PvEBP-2 and PvDBPII as compared to captive animals from Indaial/SC. This finding of high levels of antibodies in free-living animals can be attributed to the forest ecosystem of the region and closer proximity of free-living animals and anopheline species, which are responsible for the parasite transmission (Marrelli et al., 2007). No significant differences were found in the percentage of responders neither in levels of antibodies when were compared males and females NP, suggesting similar exposure to malaria infection. In humans, men from some endemic areas showed higher responsivity against malaria antigens, probably due to their occupation which might increases exposure to the malaria transmission (Del Giudice et al., 1990; Kale et al., 2019). The humoral response against CSP peptides from different *Plasmodium* species was higher in adults than non-adult animals, suggesting that the humoral immune response is correlated with the cumulative exposure to malaria infection throughout their lives, as demonstrated in humans (Tapchaisri et al., 1985; Esposito et al., 1986; Griffin et al., 2015). No significant differences were observed in the frequency of responders against erythrocytic antigens of *P. vivax* comparing adult and non-adult animals. However, the levels of antibodies against PvEBP-2 and PvDBPII were significantly higher in adults, suggesting that the magnitude of humoral response depends on the repetitive cycles of erythrocytic stages. Since this study was not designed for comparison between adult and non-adult monkeys and because of the difficult to access non-adult animals, the number of adults is 5 times higher than non-adult animals. Therefore, these findings must be carefully analyzed. Although of this limitation, at least one comparison had a strong statistic power (DBP-II, 0.847), confirming that in fact adults might have stronger response for specific antigens. Moreover, in humans, the humoral immune response against erythrocytic antigens also increases with age due to repeated exposure to malaria parasites (Del Giudice et al., 1990; Kano et al., 2012; Hester et al., 2013; Griffin et al., 2015; Stanisic et al., 2015).

The level of antibodies and rate of seroprevalence against Pb/PmCSP were higher in NP infected by *P. brasilianum/P. malariae*. The seroprevalence against PvEBP-2 and PvDBPII and level of antibodies against DBPII were higher in *P. simium* infected NP. Similar results were observed in Papua New Guinean children with a concurrent *P. vivax* infection which had significantly higher IgG levels against PvDBPII and PvEBP-2 (He et al., 2019). The absence of correlation between PCR positivity and antibody responses against the other antigens PvCSP and PvAMA-1 observed here was also previously observed in humans (Burkot et al., 1989; Kilombero Malaria Project, 1992; Campo et al., 2011; Pereira et al., 2018). However, in PCR negatives monkeys, we cannot rule out the possibility of parasites being in hematopoietic niches, which would explain the absence of parasites in the peripheral circulation (Obaldia et al., 2018; Silva-Filho et al., 2020).

In Indaial and Joinville we sampled only *Alouatta guariba clamitans*, but in Guapimirim we included primates belonging to all Platyrrhini families. Thus, the evaluation of the immune responses among animals from Guapimirim provide an excellent opportunity to compare how each monkey family responds against *Plasmodium* sp. antigens under the same epidemiological conditions. Overall, most monkey members of the five families had IgG antibodies against at least one CSP repeat, 100% of Atelidae and Pithecidae, 92% of Cebidae, and 50% of Callitrichidae. The reactivity was homogeneous across different peptides, i.e., most animals responded to all three CSP peptides. However, reactivity against *P. vivax* erythrocytic antigens was observed for some antigens but not others. IgG antibodies against at least one of these antigens was detected in all specimens of Pitheciidae, in half of Atelidae specimens and half of Callitrichidae specimens, but in only 17% of Cebidae specimens. *Alouatta guariba clamitans* of Atelidae family, from Atlantic Forest of São Paulo and Santa Catarina states, previously showed antibodies against CSP, MSP1_19_ and crude blood stage antigens (by IFA) of *P. vivax*, *P. falciparum* and *P. malariae/P. brasilianum* (Duarte et al., 2006; Yamasaki et al., 2011; Monteiro et al., 2020). Animals of Cebidae family previously showed antibodies against CSP peptides but did not against erythrocytic antigens, and animals of Callitrichidae family did not have antibodies against either pre-erythrocytic or erythrocytic antigens (MSP1_19_ and crude blood stage antigens – IFA) for different *Plasmodium* species (Duarte et al., 2006; Yamasaki et al., 2011; Monteiro et al., 2020). Therefore, all families seem to be exposed to malaria transmission, but the establishment of blood malaria infection might only occur in Atelidae family, which could be the potential malaria reservoirs. Although, some species of Pitheciidae family also showed antibody response against erythrocytic antigens, they might not be important reservoirs in the Atlantic Forest because of the two studied species only *Callicebus personatus* is native of this region. Moreover, this conclusion must be taken with caution since some families were very poorly studied, in number of specimens and species. Interestingly, *Alouatta guariba clamintas* and *Brachyteles arachnoides*, species initially described infected by *P. simium* (Deane et al., 1968; Deane, 1992), showed antibodies against CSP and also erythrocytic antigens of *Plasmodium* sp. *Alouatta* is the genus with highest frequency of *Plasmodium* infection in the Atlantic forest (Deane, 1992; Duarte et al., 2006; Yamasaki et al., 2011; de Alvarenga et al., 2015; Alvarenga et al., 2017; Abreu et al., 2019). In conjunction with our findings of humoral immune response, this genus, particularly *A. g. clamitans* was suggested as the main potential reservoir of malaria in the Atlantic Forest (Abreu et al., 2019). However, the detection of gametocytes (sexual stage of parasite) and transmission experiments are still needed for evaluation of the potential for transmission and confirmation of their role as malaria reservoirs. Therefore, the growth of human intrusion in forest areas reinforces the need of the inclusion of the Atlantic Forest as malaria transmission area. In this region, malaria prevention and control depend on environmental education strategies for the local population and tourists, and epidemiological surveillance of zoonotic malaria.

Our results show that both male and females of neotropical primates from Brazilian Atlantic Forest have had contact with *Plasmodium* sporozoites. However, it seems that mainly adults of some species of Atelidae family constantly exposed to malaria transmission might develop an erythrocytic infection, potentially serving as reservoirs of malaria. Taken together, these data suggest that immune response recorded in naturally infected monkeys is similar to that reported in human exposed to malaria in endemic areas (Kano et al., 2012; França et al., 2017; Pereira et al., 2018; Kale et al., 2019).

## Data Availability Statement

The original contributions presented in the study are included in the article/**Supplementary Material**. Further inquiries can be directed to the corresponding author.

## Ethics Statement

The animal study was reviewed and approved by Fiocruz Research Ethical Committee (CEUA license L037/2016) and by the Brazilian Ministry of Environment (SISBIO numbers 43375-4, 43375-6, 54707-137362-2, 52472-1, 28953-1).

## Author Contributions

CA, LC, JO, and GA designed and conception the study. GA, DA, APC, JS, AN, AP, SM, HP, VP, and ZH were responsible for sample collection. LM, HL, FK, IS, FN, JA, and JO were responsible for the production of *Plasmodium* proteins and peptides. GA, DA, MC, JS-A, and JO performed the experiments. GA, DA, TS, FK, JO, LC, CD-R, and CA analyzed and interpreted data. GA, DA, and CA wrote the first draft. All authors contributed to the article and approved the submitted version.

## Funding

This study was supported by Conselho Nacional de Desenvolvimento Científico e Tecnológia (CNPq - Grant nos. 457274/2014-0, 310477/2017-4), Fundação de Amparo a Pesquisa do estado de Minas Gerais (FAPEMIG – Grant no. CBB-APQ-02620-15), Fiocruz Inova Grant for innovative products (VPPIS-004-FIO-18-16), and the Secretary for Health Surveillance of the Brazilian Ministry of Health (IOC-017-FIO-17 and IOC-028-FIO-18). This study was partially supported by the Coordenação de Aperfeiçoamento de Pessoal de Nível Superior – Brasil (CAPES) – Finance Code 001.

## Conflict of Interest

The authors declare that the research was conducted in the absence of any commercial or financial relationships that could be construed as a potential conflict of interest.

The reviewer TO declared a shared affiliation, with no collaboration, with one of the authors, IS, to the handling editor at the time of review.

## Abbreviations

CEPESBI, Centre for Biological Research of Indaial; CPRJ, Primate Centre of Rio de Janeiro; CSP, Circumsporozoite protein; ELISA, Enzyme-linked immunosorbent assay; NP, Neotropical primates; OD, Optical density; Pb/PmCSP, Circumsporozoite protein repeats of *P. brasilianum/P.malariae*; PCR, Polymerase Chain Reaction; PfCSP, Circumsporozoite protein repeats of *P. falciparum*; PvAMA-1, *P. vivax* Apical Membrane antigen – 1; PvCSP, Circumsporozoite protein repeats of *P. vivax*; PvDBPII, *P. vivax* Duffy Binding Protein region II, PvEBP-2: *P. vivax Erythrocyte Binding* Protein 2*;* RI, Reactivity index; SD, Standard deviation.
