# Supplementary figures and images for: Profiling Humoral Immune Response Against Pre-Erythrocytic and Erythrocytic Antigens of Malaria Parasites Among Neotropical Primates in the Brazilian Atlantic Forest
Source: Front Cell Infect Microbiol. 2021 May 13;11:678996. doi: 10.3389/fcimb.2021.678996 (PMC8155606; doi:10.3389/fcimb.2021.678996)

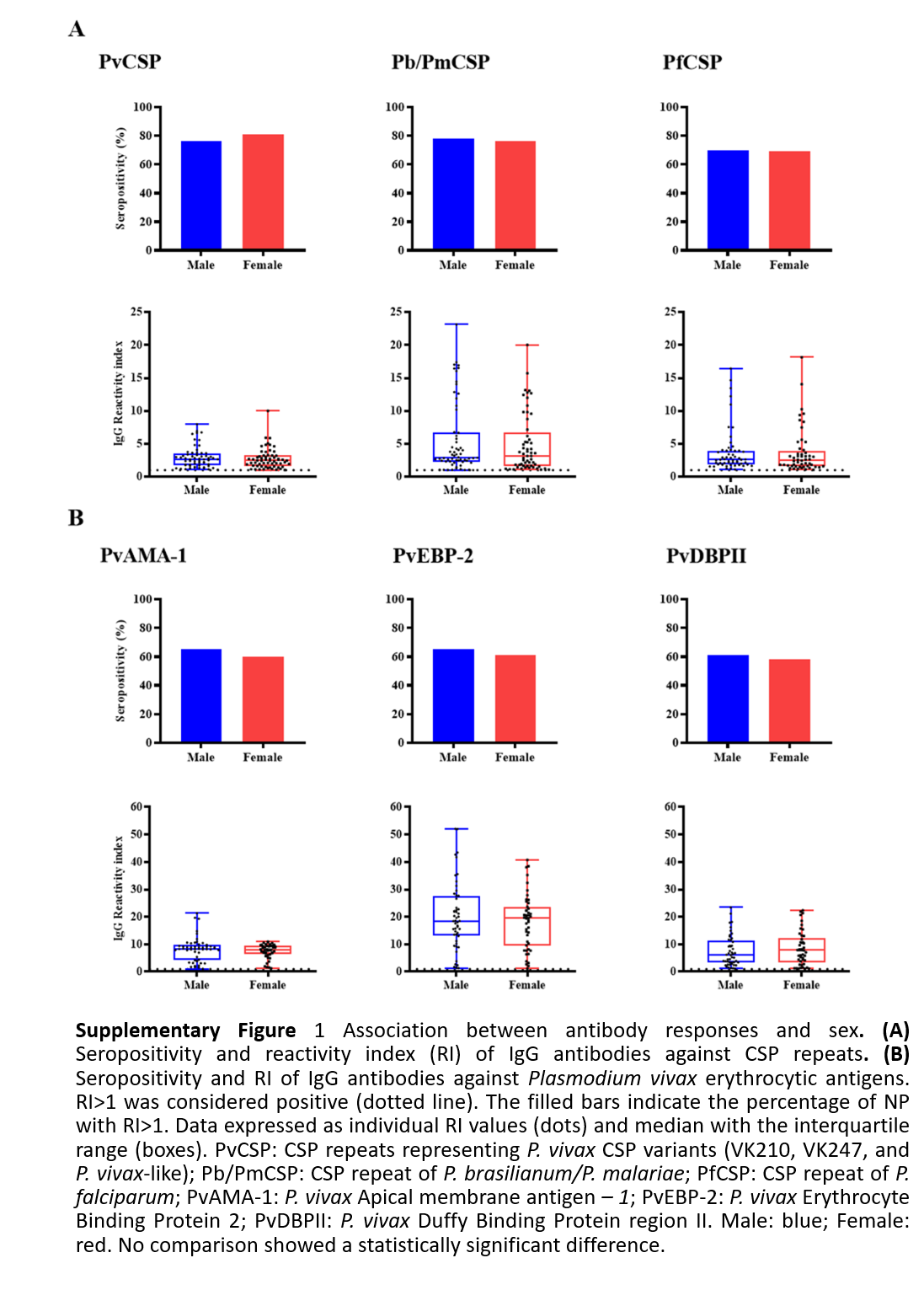

Supplement: Supplementary file 1 [file Image_1.tif]

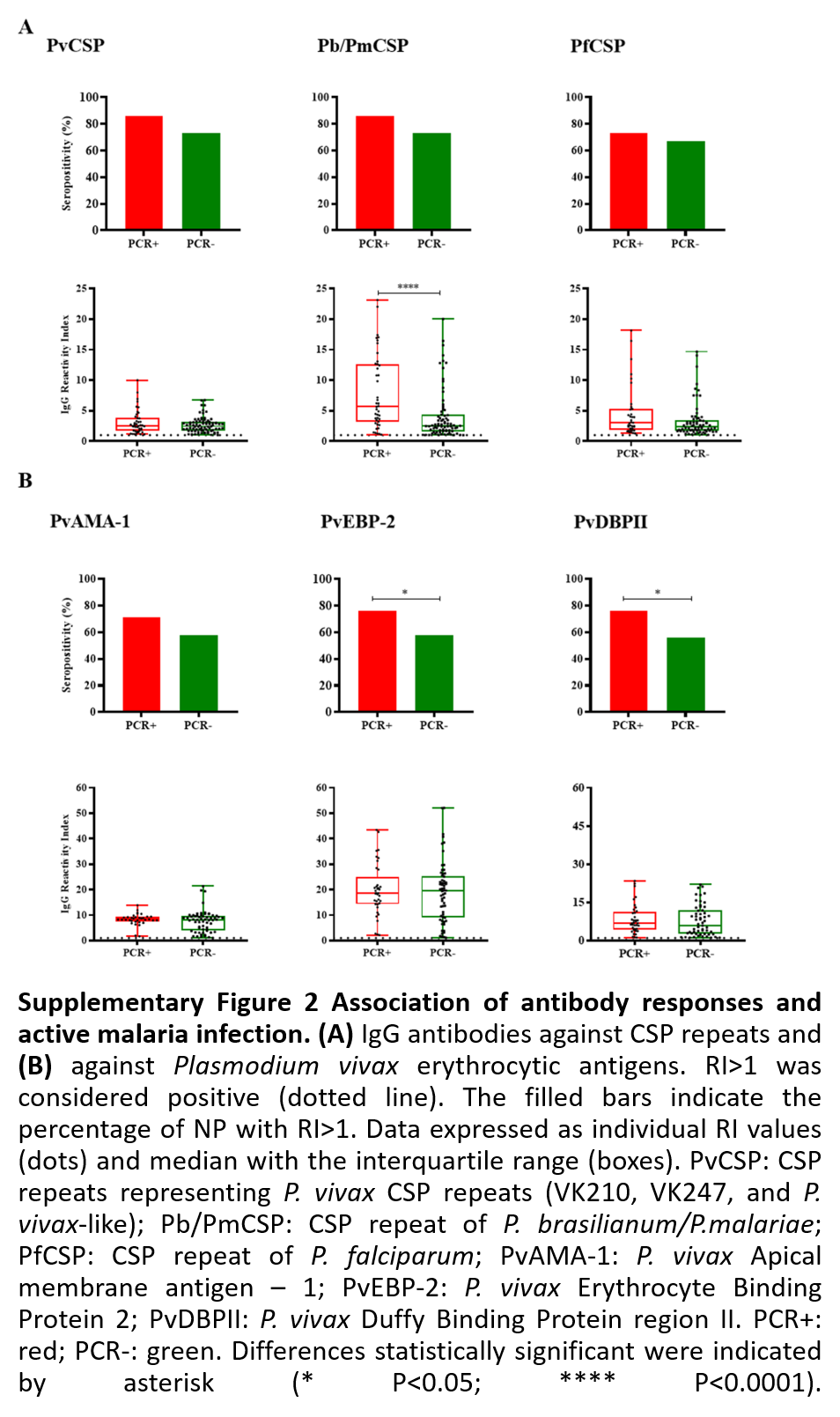

Supplement: Supplementary file 2 [file Image_2.tif]
