# Supplementary material for: Profiling Humoral Immune Response Against Pre-Erythrocytic and Erythrocytic Antigens of Malaria Parasites Among Neotropical Primates in the Brazilian Atlantic Forest
Source: Front Cell Infect Microbiol. 2021 May 13;11:678996. doi: 10.3389/fcimb.2021.678996 (PMC8155606; doi:10.3389/fcimb.2021.678996)
Supplement: Supplementary file 3 [file Table_1.docx]

| **Study area** | **Animal code** | **Family** | **Species** | **Sex^a^** | **Age^b^** | **Captive/ Free-living** | **Diagnosis of infection^c^** |
| --- | --- | --- | --- | --- | --- | --- | --- |
| Indaial/SC | **BL 1** | Atelidae | *Alouatta g. clamitans* | M | A | Captive | N |
| Indaial/SC | **BL 2** |  | *Alouatta g. clamitans* | M | A | Captive | N |
| Indaial/SC | **BL10** |  | *Alouatta g. clamitans* | M | A | Captive | Ps |
| Indaial/SC | **BL11** |  | *Alouatta g. clamitans* | F | A | Captive | N |
| Indaial/SC | **BL12** |  | *Alouatta g. clamitans* | M | A | Captive | N |
| Indaial/SC | **BL13** |  | *Alouatta g. clamitans* | F | A | Captive | N |
| Indaial/SC | **BL14** |  | *Alouatta g. clamitans* | M | A | Captive | N |
| Indaial/SC | **BL15** |  | *Alouatta g. clamitans* | M | NA | Captive | N |
| Indaial/SC | **BL16** |  | *Alouatta g. clamitans* | M | A | Captive | N |
| Indaial/SC | **BL17** |  | *Alouatta g. clamitans* | F | A | Captive | N |
| Indaial/SC | **BL18** |  | *Alouatta g. clamitans* | M | A | Captive | N |
| Indaial/SC | **BL22** |  | *Alouatta g. clamitans* | M | A | Captive | N |
| Indaial/SC | **BL25** |  | *Alouatta g. clamitans* | M | A | Captive | N |
| Indaial/SC | **BL30** |  | *Alouatta g. clamitans* | F | A | Captive | N |
| Indaial/SC | **BL31** |  | *Alouatta g. clamitans* | F | A | Captive | N |
| Indaial/SC | **BL32** |  | *Alouatta g. clamitans* | F | A | Captive | N |
| Indaial/SC | **BL33** |  | *Alouatta g. clamitans* | F | A | Captive | N |
| Indaial/SC | **BL34** |  | *Alouatta g. clamitans* | F | A | Captive | N |
| Indaial/SC | **BL35** |  | *Alouatta g. clamitans* | F | A | Captive | N |
| Indaial/SC | **BL36** |  | *Alouatta g. clamitans* | F | A | Captive | N |
| Indaial/SC | **BL37** |  | *Alouatta g. clamitans* | M | A | Captive | N |
| Indaial/SC | **BL38** |  | *Alouatta g. clamitans* | M | A | Captive | N |
| Indaial/SC | **BL39** |  | *Alouatta g. clamitans* | M | A | Captive | N |
| Indaial/SC | **BL40** |  | *Alouatta g. clamitans* | M | A | Captive | N |
| Indaial/SC | **BL41** |  | *Alouatta g. clamitans* | F | A | Captive | N |
| Indaial/SC | **BL42** |  | *Alouatta g. clamitans* | M | A | Captive | N |
| Indaial/SC | **BL43** |  | *Alouatta g. clamitans* | M | A | Captive | N |
| Indaial/SC | **BL44** |  | *Alouatta g. clamitans* | F | A | Captive | N |
| Indaial/SC | **BL45** |  | *Alouatta g. clamitans* | M | A | Captive | N |
| Indaial/SC | **BL46** |  | *Alouatta g. clamitans* | M | A | Captive | N |
| Indaial/SC | **BL47** |  | *Alouatta g. clamitans* | M | NA | Captive | N |
| Indaial/SC | **BL48** |  | *Alouatta g. clamitans* | F | A | Captive | N |
| Indaial/SC | **BL49** |  | *Alouatta g. clamitans* | F | A | Captive | N |
| Indaial/SC | **BL50** |  | *Alouatta g. clamitans* | M | A | Captive | N |
| Indaial/SC | **BL51** |  | *Alouatta g. clamitans* | F | A | Captive | N |
| Indaial/SC | **BL62** |  | *Alouatta g. clamitans* | M | A | Free-living | Ps |
| Indaial/SC | **BL64** |  | *Alouatta g. clamitans* | F | NA | Free-living | Ps |
| Indaial/SC | **BL68** |  | *Alouatta g. clamitans* | F | A | Free-living | N |
| Indaial/SC | **BL69** |  | *Alouatta g. clamitans* | F | A | Free-living | Ps |
| Indaial/SC | **BL77** |  | *Alouatta g. clamitans* | M | A | Captive | N |
| Indaial/SC | **BL83** |  | *Alouatta g. clamitans* | F | A | Free-living | N |
| Indaial/SC | **BL84** |  | *Alouatta g. clamitans* | M | A | Free-living | N |
| Indaial/SC | **BL85** |  | *Alouatta g. clamitans* | F | A | Free-living | N |
| Indaial/SC | **BL86** |  | *Alouatta g. clamitans* | F | A | Free-living | N |
| Indaial/SC | **BL87** |  | *Alouatta g. clamitans* | F | A | Free-living | N |
| Indaial/SC | **BL90** |  | *Alouatta g. clamitans* | M | A | Captive | N |
| Indaial/SC | **BL91** |  | *Alouatta g. clamitans* | F | A | Free-living | N |
| Indaial/SC | **BL92** |  | *Alouatta g. clamitans* | F | A | Free-living | N |
| Indaial/SC | **Bugio SARA** |  | *Alouatta g. clamitans* | M | A | Free-living | N |
| Indaial/SC | **Bugio 2** |  | *Alouatta g. clamitans* | F | NA | captive | N |
| Indaial/SC | **Bugio 3** |  | *Alouatta g. clamitans* | F | NA | captive | Ps |
| Indaial/SC | **Bugio 4** |  | *Alouatta g. clamitans* | M | A | captive | N |
| Indaial/SC | **Bugio 5** |  | *Alouatta g. clamitans* | F | NA | captive | N |
| Indaial/SC | **Bugio 6** |  | *Alouatta g. clamitans* | M | A | captive | N |
| Indaial/SC | **Bugio 7** |  | *Alouatta g. clamitans* | F | A | captive | Ps |
| Indaial/SC | **Bugio 8** |  | *Alouatta g. clamitans* | M | A | captive | N |
| Indaial/SC | **Bugio 9** |  | *Alouatta g. clamitans* | M | NA | captive | N |
| Indaial/SC | **Bugio 10** |  | *Alouatta g. clamitans* | F | NA | captive | N |
| Indaial/SC | **Bugio 12** |  | *Alouatta g. clamitans* | F | A | captive | Ps |
| Indaial/SC | **Bugio 13** |  | *Alouatta g. clamitans* | M | NA | captive | N |
| Indaial/SC | **Bugio 14** |  | *Alouatta g. clamitans* | M | NA | captive | N |
| Indaial/SC | **Bugio 15** |  | *Alouatta g. clamitans* | F | A | captive | N |
| Indaial/SC | **Bugio 19** |  | *Alouatta g. clamitans* | M | A | captive | N |
| Indaial/SC | **Bugio 20** |  | *Alouatta g. clamitans* | F | NA | captive | Ps |
| Indaial/SC | **Bugio 21** |  | *Alouatta g. clamitans* | M | NA | captive | Ps |
| Indaial/SC | **Bugio 22** |  | *Alouatta g. clamitans* | F | A | captive | N |
| Indaial/SC | **Bugio 23** |  | *Alouatta g. clamitans* | M | A | captive | N |
| Indaial/SC | **Bugio 26** |  | *Alouatta g. clamitans* | M | A | captive | N |
| Indaial/SC | **Bugio 31** |  | *Alouatta g. clamitans* | M | A | captive | N |
| Indaial/SC | **Bugio 33** |  | *Alouatta g. clamitans* | F | A | captive | N |
| Indaial/SC | **Bugio 35** |  | *Alouatta g. clamitans* | M | NA | captive | N |
| Indaial/SC | **Bugio 36** |  | *Alouatta g. clamitans* | M | A | captive | N |
| Indaial/SC | **Bugio 37** |  | *Alouatta g. clamitans* | M | A | captive | Pb/Pm |
| Indaial/SC | **Massaranduba** |  | *Alouatta g. clamitans* | F | A | captive | N |
| Indaial/SC | **Bugio 11 01** |  | *Alouatta g. clamitans* | M | A | captive | N |
| Joinville/SC | **1 PCBR** |  | *Alouatta g. clamitans* | F | A | Free-living | Mixed |
| Joinville/SC | **2 PCBR** |  | *Alouatta g. clamitans* | F | A | Free-living | Mixed |
| Joinville/SC | **3 PCBR** |  | *Alouatta g. clamitans* | M | A | Free-living | Mixed |
| Joinville/SC | **4 PCBR** |  | *Alouatta g. clamitans* | M | A | Free-living | Mixed |
| Joinville/SC | **5 PCBR** |  | *Alouatta g. clamitans* | M | A | Free-living | Mixed |
| Joinville/SC | **6 PCBR** |  | *Alouatta g. clamitans* | F | A | Free-living | N |
| Joinville/SC | **9 PCBR** |  | *Alouatta g. clamitans* | M | A | Free-living | Ps |
| Joinville/SC | **10 PCBR** |  | *Alouatta g. clamitans* | M | A | Free-living | Pb/Pm |
| Joinville/SC | **11 PCBR** |  | *Alouatta g. clamitans* | F | NA | Free-living | Ps |
| Joinville/SC | **12 PCBR** |  | *Alouatta g. clamitans* | F | A | Free-living | N |
| Joinville/SC | **13 PCBR** |  | *Alouatta g. clamitans* | M | A | Free-living | Pb/Pm |
| Joinville/SC | **14 PCBR** |  | *Alouatta g. clamitans* | F | A | Free-living | Mixed |
| Joinville/SC | **15 PCBR** |  | *Alouatta g. clamitans* | M | A | Free-living | Ps |
| Joinville/SC | **16 PCBR** |  | *Alouatta g. clamitans* | F | A | Free-living | N |
| Joinville/SC | **17 PCBR** |  | *Alouatta g. clamitans* | F | A | Free-living | Mixed |
| Joinville/SC | **18 PCBR** |  | *Alouatta g. clamitans* | M | A | Free-living | N |
| Joinville/SC | **19 PCBR** |  | *Alouatta g. clamitans* | F | A | Free-living | N |
| Joinville/SC | **20 PCBR** |  | *Alouatta g. clamitans* | F | NA | Free-living | Ps |
| Joinville/SC | **21 PCBR** |  | *Alouatta g. clamitans* | M | NA | Free-living | Mixed |
| Joinville/SC | **22 PCBR** |  | *Alouatta g. clamitans* | F | A | Free-living | Ps |
| Joinville/SC | **23 PCBR** |  | *Alouatta g. clamitans* | M | A | Free-living | N |
| Joinville/SC | **24 PCBR** |  | *Alouatta g. clamitans* | M | NA | Free-living | Pb/Pm |
| Joinville/SC | **25 PCBR** |  | *Alouatta g. clamitans* | F | NA | Free-living | Ps |
| Joinville/SC | **26 PCBR** |  | *Alouatta g. clamitans* | M | NA | Free-living | Mixed |
| Joinville/SC | **27 PCBR** |  | *Alouatta g. clamitans* | F | A | Free-living | Mixed |
| Joinville/SC | **28 PCBR** |  | *Alouatta g. clamitans* | F | NA | Free-living | Mixed |
| Joinville/SC | **29 PCBR** |  | *Alouatta g. clamitans* | F | A | Free-living | Mixed |
| Joinville/SC | **31 PCBR** |  | *Alouatta g. clamitans* | M | A | Free-living | Ps |
| Joinville/SC | **32 PCBR** |  | *Alouatta g. clamitans* | F | A | Free-living | Ps |
| Joinville/SC | **33 PCBR** |  | *Alouatta g. clamitans* | F | A | Free-living | Ps |
| Joinville/SC | **35 PCBR** |  | *Alouatta g. clamitans* | M | NA | Free-living | N |
| Joinville/SC | **36 PCBR** |  | *Alouatta g. clamitans* | F | A | Free-living | N |
| Joinville/SC | **37 PCBR** |  | *Alouatta g. clamitans* | M | A | Free-living | N |
| Joinville/SC | **41 PCBR** |  | *Alouatta g. clamitans* | M | A | Free-living | N |
| Joinville/SC | **43 PCBR** |  | *Alouatta g. clamitans* | M | A | Free-living | Mixed |
| Joinville/SC | **45 PCBR** |  | *Alouatta g. clamitans* | F | A | Free-living | N |
| Joinville/SC | **46 PCBR** |  | *Alouatta g. clamitans* | M | A | Free-living | N |
| Joinville/SC | **47 PCBR** |  | *Alouatta g. clamitans* | F | A | Free-living | N |
| Joinville/SC | **49 PCBR** |  | *Alouatta g. clamitans* | F | A | Free-living | Mixed |
| Guapimirim/RJ | **2443** |  | *Alouatta g. clamitans* | M | A | Captive | Pb/Pm |
| Guapimirim/RJ | **2576** |  | *Alouatta Caraya* | M | A | Captive | N |
| Guapimirim/RJ | **2049** |  | *Brachyteles arachnoides* | F | A | Captive | N |
| Guapimirim/RJ | **2263** |  | *Brachyteles arachnoides* | M | A | Captive | N |
| Guapimirim/RJ | **3078** |  | *Brachyteles arachnoides* | M | A | Captive | N |
| Guapimirim/RJ | **2619** |  | *Ateles paniscus* | F | A | Captive | N |
| Guapimirim/RJ | **3636** |  | *Alouatta g clamitans* | M | A | Captive | N |
| Guapimirim/RJ | **3622** |  | *Alouatta g clamitans* | F | A | Captive | Pb/Pm |
| Guapimirim/RJ | **2203** | Aotidae | *Aotus nigriceps* | M | A | Captive | Pb/Pm |
| Guapimirim/RJ | **2299** | Callitrichidae | *Saguinus midas* | M | A | Captive | N |
| Guapimirim/RJ | **2359** |  | *Leontopithecus chrysomelas* | F | A | Captive | N |
| Guapimirim/RJ | **2435** |  | *Leontopithecus rosalia* | M | A | Captive | N |
| Guapimirim/RJ | **2390** |  | *Mico humeralifer* | M | A | Captive | Pb/Pm |
| Guapimirim/RJ | **1624** | Cebidae | *Sapajus xanthosternos* | M | A | Captive | N |
| Guapimirim/RJ | **2005** |  | *Sapajus xanthosternos* | F | A | Captive | Pb/Pm |
| Guapimirim/RJ | **2046** |  | *Sapajus xanthosternos* | F | A | Captive | N |
| Guapimirim/RJ | **2098** |  | *Sapajus xanthosternos* | F | A | Captive | Ps |
| Guapimirim/RJ | **2110** |  | *Sapajus robustus* | F | A | Captive | N |
| Guapimirim/RJ | **2125** |  | *Sapajus xanthosternos* | F | A | Captive | N |
| Guapimirim/RJ | **2135** |  | *Sapajus xanthosternos* | F | A | Captive | N |
| Guapimirim/RJ | **2183** |  | *Sapajus xanthosternos* | F | A | Captive | N |
| Guapimirim/RJ | **2207** |  | *Sapajus robustus* | M | A | Captive | N |
| Guapimirim/RJ | **2297** |  | *Sapajus robustus* | F | A | Captive | N |
| Guapimirim/RJ | **2324** |  | *Sapajus xanthosternos* | F | A | Captive | Ps |
| Guapimirim/RJ | **2360** |  | *Sapajus robustus* | F | A | Captive | N |
| Guapimirim/RJ | **2388** |  | *Sapajus xanthosternos* | M | A | Captive | N |
| Guapimirim/RJ | **2392** |  | *Sapajus xanthosternos* | F | A | Captive | N |
| Guapimirim/RJ | **2501** |  | *Cebus sp.** | M | A | Captive | N |
| Guapimirim/RJ | **2503** |  | *Cebus sp.** | F | A | Captive | Ps |
| Guapimirim/RJ | **2504** |  | *Cebus sp*.* | F | A | Captive | N |
| Guapimirim/RJ | **2536** |  | *Sapajus xanthosternos* | M | A | Captive | N |
| Guapimirim/RJ | **2559** |  | *Cebus sp.** | F | A | Captive | Ps |
| Guapimirim/RJ | **2564** |  | *Sapajus robustus* | M | A | Captive | Ps |
| Guapimirim/RJ | **2456** |  | *Sapajus robustus* | M | A | Captive | N |
| Guapimirim/RJ | **2539** |  | *Sapajus xanthosternus* | M | A | Captive | N |
| Guapimirim/RJ | **2592** |  | *Sapajus xanthosternus* | M | A | Captive | N |
| Guapimirim/RJ | **2208** |  | *Sapajus robustus* | F | A | Captive | N |
| Guapimirim/RJ | **2466** | Pithecidae | *Callicebus personatus* | F | A | Captive | Pb/Pm |
| Guapimirim/RJ | **2302** |  | *Cacajao melanocephalus* | F | A | Captive | Ps |
| Guapimirim/RJ | **2620** |  | *Cacajao melanocephalus* | F | A | Captive | Pb/Pm |

**Supplementary Table 1. Characteristics of studied neotropical primates from three areas of Atlantic Forest**. a: F= Female; M= Male; b: age estimated according to (Carpenter, 1965) A= Adult (including subadult); NA= Non Adults (juvenile); c: Molecular diagnosis of *Plasmodium* sp. were performed by (Snounou et al., 1993; de Alvarenga et al., 2018). Mixed= mixed infection (*P. simium*+ *P. brasilianum/P. malariae*), Pb/Pm= *P. brasilianum/P. malariae*; Ps = *P. simium,* N= negative.
